# WRKY76 is a rice transcriptional repressor playing opposite roles in blast disease resistance and cold stress tolerance

**DOI:** 10.1093/jxb/ert298

**Published:** 2013-09-16

**Authors:** Naoki Yokotani, Yuko Sato, Shigeru Tanabe, Tetsuya Chujo, Takafumi Shimizu, Kazunori Okada, Hisakazu Yamane, Masaki Shimono, Shoji Sugano, Hiroshi Takatsuji, Hisatoshi Kaku, Eiichi Minami, Yoko Nishizawa

**Affiliations:** ^1^Disease Resistant Crops Research Unit, GMO Research Center, National Institute of Agrobiological Sciences, 2-1-2 Kannondai, Tsukuba, Ibaraki 305-8602, Japan; ^2^Biotechnology Research Center, The University of Tokyo, 1-1-1 Yayoi, Bunkyo-ku, Tokyo 113-8657, Japan; ^3^Department of Biosciences, Teikyo University, 1-1 Toyosatodai, Utsunomiya, Tochigi 320-8551, Japan; ^4^Sakata Seed Corporation, Kimitsu Research Station, 358 Uchikoshi, Sodegaura, Chiba 299-0217, Japan

**Keywords:** Blast disease resistance, cold stress, phytoalexin, rice, transcriptional repressor, WRKY.

## Abstract

*OsWRKY76* encodes a group IIa WRKY transcription factor of rice. The expression of *OsWRKY76* was induced within 48h after inoculation with rice blast fungus (*Magnaporthe oryzae*), and by wounding, low temperature, benzothiadiazole, and abscisic acid. Green fluorescent protein-fused OsWRKY76 localized to the nuclei in rice epidermal cells. OsWRKY76 showed sequence-specific DNA binding to the W-box element *in vitro* and exhibited W-box-mediated transcriptional repressor activity in cultured rice cells. Overexpression of *OsWRKY76* in rice plants resulted in drastically increased susceptibility to *M. oryzae*, but improved tolerance to cold stress. Microarray analysis revealed that overexpression of *OsWRKY76* suppresses the induction of a specific set of *PR* genes and of genes involved in phytoalexin synthesis after inoculation with blast fungus, consistent with the observation that the levels of phytoalexins in the transgenic rice plants remained significantly lower than those in non-transformed control plants. Furthermore, overexpression of *OsWRKY76* led to the increased expression of abiotic stress-associated genes such as peroxidase and lipid metabolism genes. These results strongly suggest that *OsWRKY76* plays dual and opposing roles in blast disease resistance and cold tolerance.

## Introduction

Plant growth is greatly affected by various types of stresses such as pathogen attacks, insect herbivory, and abiotic environmental stresses including high salinity, drought, and excessive temperature. In order to maintain growth and productivity, plants have developed adaptive responses to these stresses. These responses are triggered by environmental cues, and the transduction of these stress signals results in the regulation of a large number of stress-associated genes (Singh *et al.*, 2002). Biotic and abiotic stress signalling pathways have been considered to form a complicated network of synergistic and antagonistic interactions (Fujita *et al.*, 2006; Sharma *et al.*, 2013). A plant hormone, abscisic acid (ABA), is involved in responses to abiotic stresses such as drought, low temperature, and high salinity stress (Yamaguchi-Shinozaki and Shinozaki, 2006). Salicylic acid (SA) plays a positive role in resistance against biotrophic and hemibiotrophic pathogens (Robert-Seilaniantz *et al.*, 2007; Thaler *et al.*, 2012). Other plant hormones, such as jasmonic acid (JA), ethylene, and brassinosteroid, also participate in the processes of biotic and abiotic stress responses (Mauch-Mani and Mauch, 2005; Yamaguchi-Shinozaki and Shinozaki, 2006).

Rice blast fungus (*Magnaporthe oryzae*, Oryzae isolate) is a hemibiotrophic pathogen that causes blast disease in rice, which accounts for major losses in the global yield of rice (Talbot, 2003). In rice, SA signalling positively regulates blast disease resistance; application of benzothiadiazole (BTH), a chemical activator of SA signalling, induces resistance in rice against *M. oryzae* (Schweizer *et al.*, 1999). On the other hand, ABA interacts antagonistically with the SA signalling pathway in the interaction between rice and *M. oryzae* (Jiang *et al.*, 2010), which might cause low temperature-induced blast disease susceptibility (Koga *et al.*, 2004). A wide range of transcriptional alterations of gene expression occurs when the rice interacts with the fungus. Previous studies have identified several families of transcription factors (TFs) that are involved in responses to infections by blast fungus. They are ERF/AP2 (Liu *et al.*, 2012), NAC (Nakashima *et al*., 2006), bHLH (Kim *et al.*, 2012), and WRKY (Pandey and Somssich, 2009).

WRKY TFs are plant-specific families of zinc finger transcription factors that are characterized by a conserved DNA-binding WRKY domain. Most WRKY TFs bind to a consensus *cis*-element termed the W-box (TTGACT/C), which is found in the promoters of many defence-associated genes, and regulates their transcription (Eulgem *et al.*, 2000; Rushton *et al.*, 2010). In rice, >100 WRKY genes have been identified in the genome (Xie *et al.*, 2005; Ross *et al.*, 2007), and at least 20 WRKY genes are transcriptionally regulated in response to *M. oryzae* inoculation, implying that they are important for the defence response (Ryu *et al.*, 2006; Bagnaresi *et al.*, 2012). In particular, *OsWRKY45* has been demonstrated to play a crucial role in mediating BTH-induced resistance to *M. oryzae*, and *Xanthomonas oryzae* pv. *oryzae*, the bacterial causal agent of rice leaf blight (Shimono *et al.*, 2007, 2012). OsWRKY45 has transcriptional activator activity and is involved in the up-regulation of various defence-associated genes. Similarly, other WRKY domain-containing transcriptional activators, including *OsWRKY53* (Chujo *et al.*, 2007), *OsWRKY31* (Zhang *et al.*, 2008), and *OsWRKY30* (Peng *et al.*, 2012), are postulated to regulate positively the defence against *M. oryzae*. In various plant species, some WRKY transcriptional activators play important roles in disease resistance, whereas some others that have transcriptional repressor activity are also transcriptionally up-regulated by pathogen infection and are considered to play a role as negative regulators of disease resistance. For example, *Arabidopsis* AtWRKY7 has repressor activity and is negatively involved in resistance to the bacterial pathogen *Pseudomonas syringae* (Kim *et al.*, 2006). In barley, *HvWRKY1* and *HvWRKY2*, which encode transcriptional repressors, are involved in the down-regulation of basal defence responses triggered by pathogen-associated molecular patterns (Shen *et al.*, 2007).

Recent studies have suggested important roles for WRKYs in abiotic stress responses (Pandey and Somssich, 2009; Rushton *et al.*, 2010). For example, *HvWRKY38* is a positive regulator of the drought stress response (Marè *et al.*, 2004; Xiong *et al.*, 2010). In *Arabidopsis*, three closely related WRKY genes, *AtWRKY18*, *40*, and *60*, are involved in both biotic and abiotic stress responses by regulating the signalling of the stress-associated plant hormones SA, JA, and ABA (Xu *et al.*, 2006; Chen *et al.*, 2010; Shang *et al.*, 2010). Another *Arabidopsis* WRKY gene, *AtWRKY25*, negatively regulates the SA-mediated defence response to *P. syringae* (Zheng *et al.*, 2007), but positively regulates responses to high salinity (Jiang and Deyholos, 2009) and high temperature (Li *et al.*, 2011) stresses. Thus, WRKY proteins are probably involved in a variety of stress responses, and their roles apparently vary depending on the stress.

*OsWRKY76* encodes a rice WRKY TF of group IIa possessing a single WRKY domain (Eulgem *et al.*, 2000; Xie *et al.*, 2005; Ross *et al.*, 2007). The expression of *OsWRKY76* is increased by *M. oryzae* inoculation and treatment with BTH, suggesting that it is also involved in the response to blast disease (Ryu *et al.*, 2006; Shimono *et al.*, 2007; Bagnaresi *et al.*, 2012). Previous studies demonstrated that the overexpression of *OsWRKY76* in rice causes reduced resistance to rice leaf blight disease (Seo *et al.*, 2011). On the basis of an interactome analysis performed using yeast two-hybrid assays, Seo *et al.* (2011) reported that *OsWRKY76* probably regulates cellular responses to both biotic and abiotic stresses. However, little is known about the detailed molecular and physiological functions of OsWRKY76. Here, it is shown that OsWRKY76 is a transcriptional repressor with DNA binding activity to the W-box element, and is localized in the nucleus. The *in vivo* biological functions of *OsWRKY76* in biotic and abiotic stresses were also investigated using transgenic rice plants overexpressing *OsWRKY76* (*W76-OX*).

## Materials and methods

### Plants and pathogens

Rice plants (*Oryza sativa* L. cv. Nipponbare) carrying the blast resistance gene *Pia* [Nipponbare (Pia)] were used in this study. Rice plants were grown in a chamber under a 14h light (28 °C) and 10h dark (24 °C) cycle in hydroponic culture, as previously described in Tanabe *et al.* (2006). *Magnaporthe oryzae* isolates Ina86-137 (MAFF 101511, race 007.0) and P91-15B (001.0) were used as virulent and avirulent strains, respectively, for Nipponbare (Pia).

### Stress and chemical treatments

Fifteen-day-old rice plants were used to examine the effects of stress and chemical treatment on the expression of *OsWRKY76*. To examine the effect of rice blast inoculation, conidia were washed and suspended at 1×10^5^ cells ml^–1^ in sterile water, and sprayed on the plants, which were then incubated at 24 °C in the dark for 24h, followed by 14h light and 10h dark cycles. For wound treatment, the fourth leaf blade was chopped into 1mm long pieces with a knife and incubated on moist filter paper at 24 °C in the dark. Cold treatment was performed by incubation at 4 °C in the dark. Chemical treatment was performed by spraying the plants with 500 μM BTH containing 0.02% (v/v) Tween-20 and 0.5% (v/v) dimethylsulphoxide (DMSO), or with 50 μM ABA containing 0.02% (v/v) Silwet L-77 and 0.1% (v/v) ethanol. The solvents of BTH or ABA were used for the mock control in each case. The fourth leaf blade was used for quantitative reverse transcription–PCT (qRT–PCR) analysis.

### Measurement of ion leakage

For measurement of ion leakage, 3cm long leaf segments were excised from the centre of each leaf blade, chopped into 5mm lengths, and incubated in 1ml of distilled water for 1h with moderate shaking. The conductivity was measured using a B-173 conductivity meter (Horiba, Kyoto, Japan) before and after the leaves were autoclaved. Ion leakage was presented as the ratio of conductivity values before and after autoclaving.

### *Transient expression of green fluorescent protein (GFP)-fused* OsWRKY76 *in rice cells*

The coding region of *OsWRKY76* was fused with the synthetic GFP gene (Niwa *et al.*, 1999), and inserted between the *Xba*I and *Sac*I sites of pBI221 (Clontech, Palo Alto, CA, USA). As a control, the 35S-GFP was used. For the transient expression assay, 1 μg of plasmid DNA was introduced into rice leaf sheath cells using the PDS-1000/He particle delivery system (BioRad, Hercules, CA, USA). As a gene expression marker and subcellular cytosol and nuclear localization marker, the plasmid containing the Discosoma red fluorescent protein (*DsRed*) gene was co-expressed. After overnight incubation in the dark, leaf sheath cells were observed under a fluorescence microscope.

### Gel mobility shift assay

The coding sequence of *OsWRKY76* was inserted between the *Bam*HI and *Hin*dIII sites of pMALc2x (New England Biolabs, Beverly, MA, USA) and introduced into *Escherichia coli* JM109. OsWRKY76 fused to maltose-binding protein (MBP) was purified using amylose resin (New England Biolabs), according to the manufacturer’s instructions. Nucleotide sequences of the probes used for DNA-binding assays are as follows: WB (5′-AACT*TTGACC*AATCTTTCAAGTA-3′) and mWB (5′-AACT*TTGAAC*AATCTTTCAAGTA-3′). The W-box or mutated W-box core sequence are underlined. A gel mobility shift assay was performed using the DIG Gel Shift Kit 2nd generation (Roche Diagnostics GmbH, Mannheim, Germany).

### Luciferase gene (LUC) reporter assay

The *GUS* gene in pBI221 was replaced with the coding sequence of *OsWRKY76*. The plasmid expressing the GAL4 DNA-binding (DB) domain was used as a control effector. The 23bp W-box-containing oligonucleotide sequence used in the gel mobility shift assay was multimerized five times (5W) to construct 35S-5W-TATA-LUC-NOS (35S-5W-LUC). In co-transfection assays, 2 μg of the reporter plasmid, 2 μg of the effector plasmid, and 0.04 μg of the internal control plasmid (pPTRL) were mixed and introduced into suspension-cultured rice cells (the Oc cell line) by particle bombardment. Transformed rice cells were incubated for 24h at 24 °C in the dark. The luciferase assay was performed using the Dual-Luciferase Reporter Assay System (Promega, Madison, WI, USA) according to the manufacturer’s instructions. Luminescence was measured using a TD20/20 luminometer (Turner Designs, Sunnyvale, CA, USA).

### RNA isolation and qRT–PCR

Total RNA was extracted using Sepasol RNA I Super (Nacalai Tesque, Kyoto, Japan). First-strand cDNA was synthesized using the PrimeScript™ RT reagent kit (TaKaRa Co., Ltd, Ohtsu, Japan). RT–PCR was performed using 1× SYBR Premix Ex Taq II (TaKaRa). The primer sequences are listed in Supplementary Table S1 available at *JXB* online. The relative levels of gene expression were quantified using MX3000P (Stratagene, La Jolla, CA, USA). The data were normalized to those of the elongation factor gene *eEF-1α* (Jain *et al.*, 2006).

### Rice transformation

For the overexpression of *OsWRKY76* in rice, the maize polyubiquitin promoter (Ubi-1), the full-length cDNA fragment of *OsWRKY76* (AK068337), and the NOS terminator were inserted between the *Hin*dIII and *Pac*I sites of the binary vector pZH1 (Shimono *et al.*, 2007), and the resultant vector was introduced into rice via *Rhizobium radiobacter* strain EHA105. Rice transformation was performed as described (Toki *et al.*, 2006).

### Fungal inoculation and microscopic observation

To evaluate disease symptoms, the fifth leaf blades were detached from rice plants at the 5.6-leaf stage and placed on moistened filter paper in Petri dishes. Washed conidia suspended at 1×10^5^ ml^–1^ in sterile water were sprayed on the leaf blades, followed by incubation at 25 °C in the dark for 24h, then under 14h light and 10h dark cycles. Blast disease development was quantified by measurement of *M. oryzae* genomic DNA (encoding 28S rRNA) relative to rice genomic DNA (encoding the *eEF-1α* gene) using quantitative genomic PCR analysis (Zellerhoff *et al.*, 2006). The primer sequences are listed in Supplementary Table S1 at *JXB* online. Data are presented relative to the value in leaves receiving immediate inoculation with conidia, which was taken as 1.

Plant response to the blast fungus at an early stage was observed in leaf sheaths under a microscope. Sheaths of the fifth leaves of rice plants at the 5.6-leaf stage were detached and inoculated with a suspension of conidia (1×10^5^ ml^–1^), and then incubated at 25 °C in the dark. After fixation in formalin-aceto-alcohol (formaldehyde–acetic acid–ethanol–water, 5:5:45:45, v/v/v/v), the level of infection was evaluated for intact appressoria under a light microscope. The samples were scored as ‘no invasion’, ‘invasion of one cell’, or ‘invasion of two or more cells’, corresponding to appressoria that penetrated into no rice cells beneath the appressorium, one cell, or more than one cell, respectively (Tanabe *et al.*, 2006).

### Determination of phytoalexins

The sheaths of the fifth leaves at the 5.6-leaf stage were detached and inoculated with Ina86-137 followed by extraction with 79% (v/v) ethanol containing 14% (v/v) water, 7% (v/v) acetonitrile, and 0.1% (v/v) acetic acid at 96 °C for 20min. Samples were analysed for the simultaneous determination of momilactones, phytocassanes, and sakuranetin using HPLC–mass spectrometry (Shimizu *et al.*, 2008). Phytoalexin levels were determined using combinations of the precursor and product ions (*m/z* 317/299 for phytocassanes A, D, and E, *m/z* 335/317 for phytocassane B, *m/z* 319/301 for phytocassane C, *m/z* 315/271 for momilactone A, *m/z* 331/269 for momilactone B, and *m/z* 287/167 for sakuranetin) in the multiple-reaction monitoring mode.

### Microarray analysis

Sheaths of the fifth leaf at the 5.6-leaf stage were used for the microarray experiment. For rice blast fungus inoculation, excised leaf sheaths were inoculated with a compatible strain of *M. oryzae* (Ina86-137) as described above and incubated at 25 °C in the dark for 36h. Cold treatment was performed by incubation at 4 °C in the dark for 36h. Leaf sheaths incubated at 25 °C in the dark for 36h were used as untreated controls. Total RNA was isolated using the RNeasay Plant Mini Kit (Qiagen, Valencia, CA, USA). Cy3-labelled complementary RNA was prepared from 200ng of total RNA and hybridized to a rice 44K oligo microarray based on the Rice Annotation Project (RAP) according to the manufacturer’s instructions (Agilent Technologies, Palo Alto, CA, USA). The slide images were scanned with a DNA microarray scanner (Agilent) using the manufacture’s Feature Extraction software.

Data were analysed using R/Bioconductor (http://www.bioconductor.org/, last accessed on 23 August 2013). Three independent leaf sheaths were used for each sample as biological replicates. After log2 transformation and global normalization, the genes for which the expression was altered significantly were selected by applying a *t*-test [one-way analysis of variance (ANOVA) Welch *t*-test, *P* = 0.01]. Data were z-score transformed across each gene and presented as a heat map represented by two-dimensional hierarchical clustering.

## Results

### *Up-regulation of* OsWRKY76 *under both biotic and abiotic stresses and by stress-related chemicals*

**Fig. 1.**
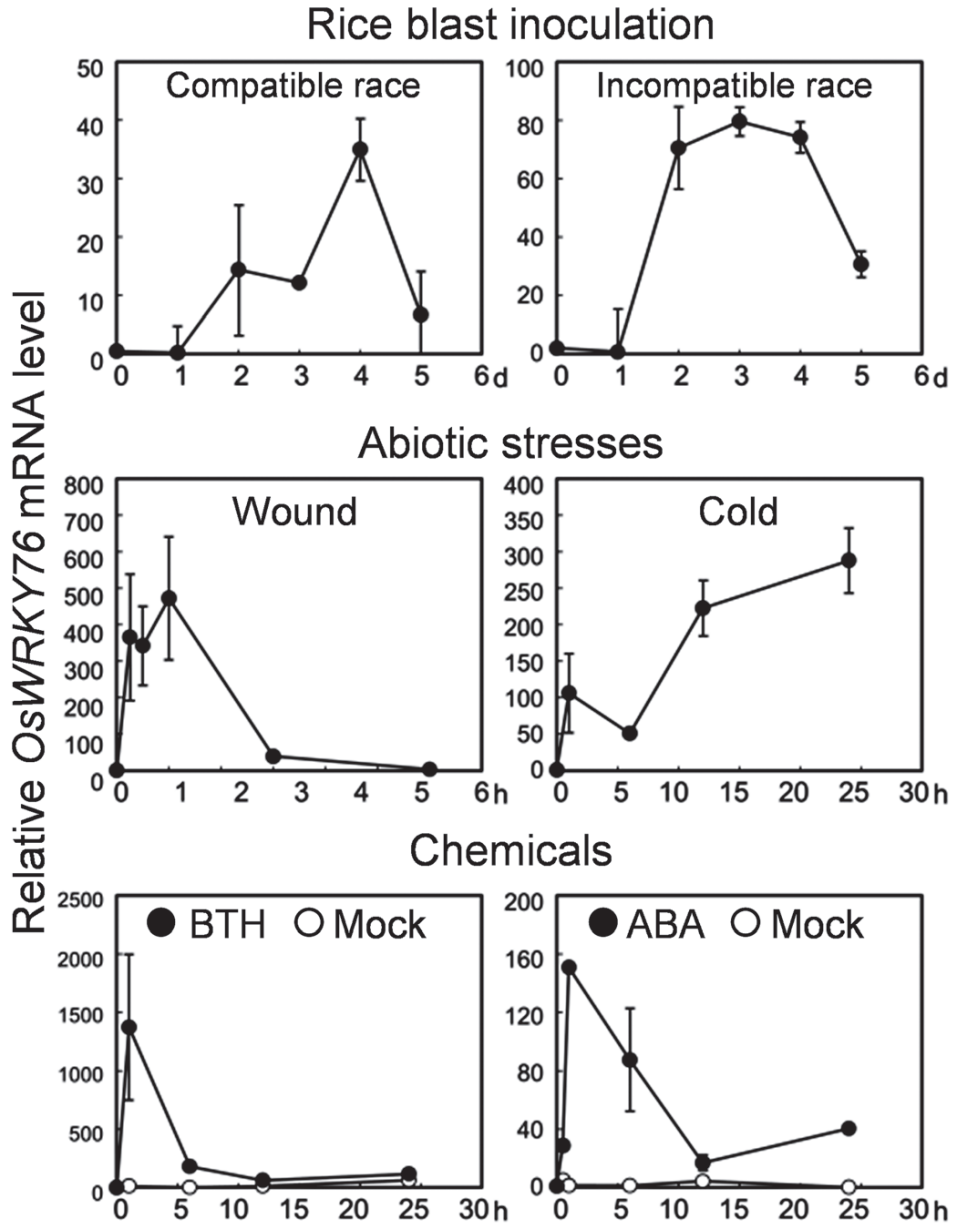
Expression of *OsWRKY76* in the response to biotic and abiotic stresses and stress-associated chemicals. *OsWRKY76* transcripts in leaf blades were measured by quantitative RT–PCR analysis. Transcription levels are expressed as the ratio to the level of transcript at 0h. Data are represented as mean values ±standard error (SE) value for three replicates.

Previous analyses demonstrated that *OsWRKY76* is up-regulated by *M. oryzae* inoculation and treatment with BTH (Ryu *et al.*, 2006; Shimono *et al.*, 2007; Bagnaresi *et al.*, 2012). To characterize the expression pattern of *OsWRKY76* further in detail, the effects of a variety of stresses and stress-related chemicals on the expression of *OsWRKY76* over time were examined by qRT–PCR analysis (Fig. 1). In untreated or mock-treated rice plants, the expression of *OsWRKY76* was nearly undetectable. The expression of *OsWRKY76* was induced by both compatible and incompatible strains of *M. oryzae* at 2 days after inoculation (dai). The levels of transcripts from *OsWRKY76* remained high until 4 dai, then decreased at 5 dai. The expression of *OsWRKY76* was rapidly induced by wounding and cold treatment. The expression of *OsWRKY76* was drastically induced by the application of BTH. ABA treatment also induced the accumulation of *OsWRKY76* transcripts within 1h. These results indicate that *OsWRKY76* is responsive to both biotic and abiotic stresses.

### Nuclear localization of OsWRKY76

**Fig. 2.**
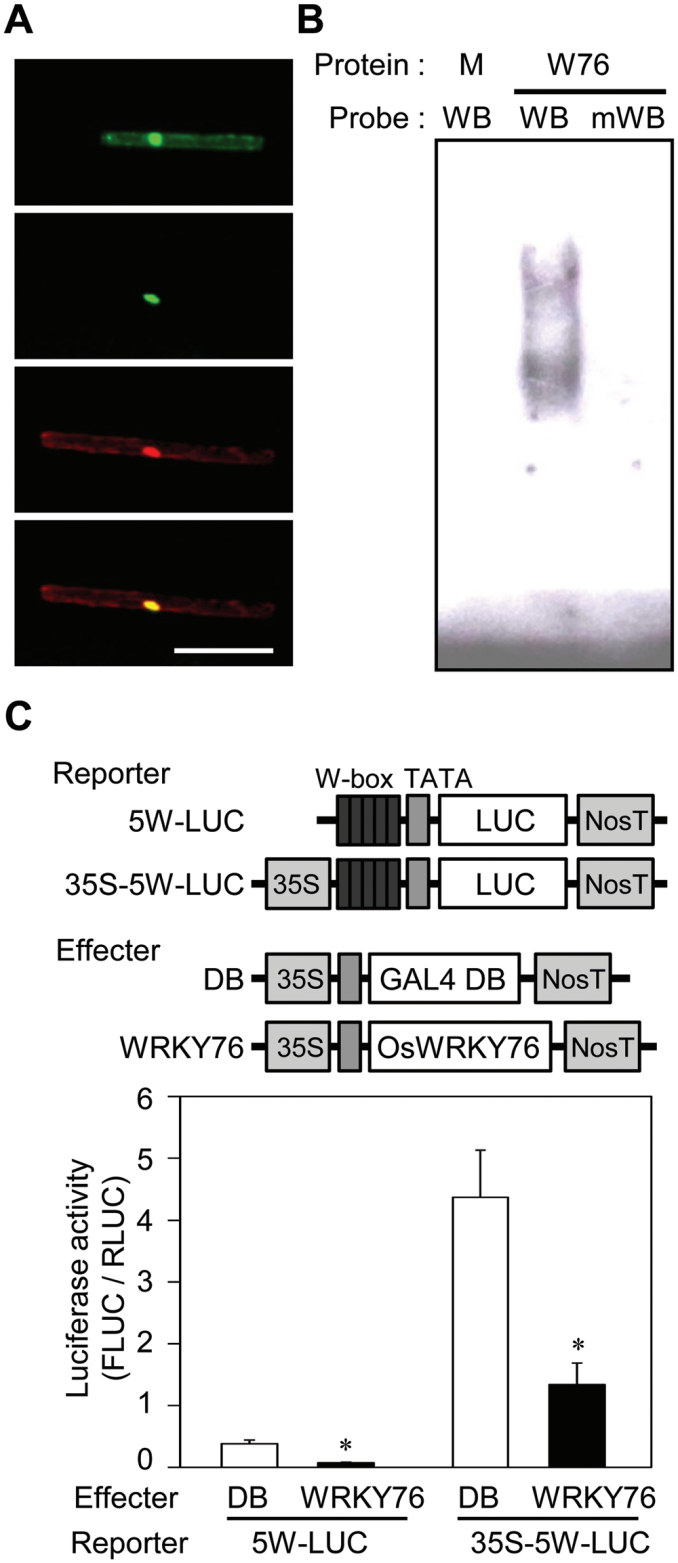
W-box-mediated transcriptional repressor activity of OsWRKY76. (A) Nuclear localization of OsWRKY76. Recombinant plasmids were transiently expressed in rice leaf sheath cells via particle bombardment and observed under a microscope. Bar = 30 μm. Top to bottom: fluorescence image of GFP control, OsWRKY76–GFP, co-expressed DsRed control, merged image of OsWRKY76–GFP and DsRed. Bar = 30 μm. (B) Binding of the OsWRKY76 protein to the W-box-containing sequence. Recombinant OsWRKY76 protein fused to MBP (W76) and MBP control (M) were used for the gel mobility shift assay. DIG-labelled W-box core sequence (WB) and mutated WB (mWB) were used as probe. (C) LUC reporter assay of W-box-mediated luciferase activity by OsWRKY76 in cultured rice cells. The data are represented as mean values ±SE for three independent experiments. Significantly lower LUC activity of OsWRKY76 compared with that of the DB control is denoted by asterisks (*P < 0.05 by *t*-test).

To investigate the subcellular localization of OsWRKY76, expression plasmids carrying GFP, GFP-fused full-length OsWRKY76 (OsWRKY76–GFP), or DsRed under the control of the *Cauliflower mosaic virus* 35S promoter were co-introduced into rice leaf sheath cells by particle bombardment. As shown in Fig. 2A, GFP and DsRed were detected in the nucleus and cytosol, whereas OsWRKY76–GFP was detected only in the nucleus. Therefore, it is concluded that the OsWRKY76 protein is targeted to the nucleus.

### Sequence-specific binding of OsWRKY76 to the W-box element

To determine the sequence-specific DNA-binding activity *in vitro*, OsWRKY76 was prepared as a fusion protein with MBP in *E. coli* and tested for its binding activity by a gel mobility shift assay. As shown in Fig. 2B, the MBP-fused OsWRKY76 protein bound to the digoxigenin (DIG)-labelled W-box core sequence (WB), but MBP control did not. Furthermore, MBP–OsWRKY76 did not bind to the mutated WB with a single base substitution. The specific interaction between MBP–OsWRKY76 and WB was effectively blocked by an excess amount of unlabelled WB but not by mutated WB (Supplementary Fig. S1 at *JXB* online). These results demonstrate that OsWRKY76 binds specifically to the W-box element.

### W-box-mediated transcriptional repressor activity of OsWRKY76

The activity of OsWRKY76 as a transcriptional regulator was tested using a chimeric effector/reporter assay (Fig. 2C). The reporter plasmids, 5W-LUC and 35S-5W-LUC, containing the firefly LUC were introduced into cultured rice cells with an effector plasmid carrying either OsWRKY76 or the GAL4 DB domain. The LUC activities of 5W-LUC and 35S-5W-LUC were considerably reduced when co-introduced with 35S-promoter-driven OsWRKY76 compared with 35S-driven GAL4-DB, indicating that OsWRKY76 acts as a transcriptional repressor via binding to the W-box.

### *Overexpression of* OsWRKY76 *increases susceptibility to the blast fungus in rice*

To characterize the biological function of *OsWRKY76*, transgenic rice plants constitutively overexpressing *OsWRKY76* under the control of the maize ubiquitin promoter were produced. Three independent lines of transgenic plants (*W76-OX#01*, *03*, and *06*) grown in a growth chamber accumulated *OsWRKY76* transcripts at a >100-fold higher level without morphological changes compared with the parental line (Supplementary Fig. S2 at *JXB* online), and were fertile.

**Fig. 3.**
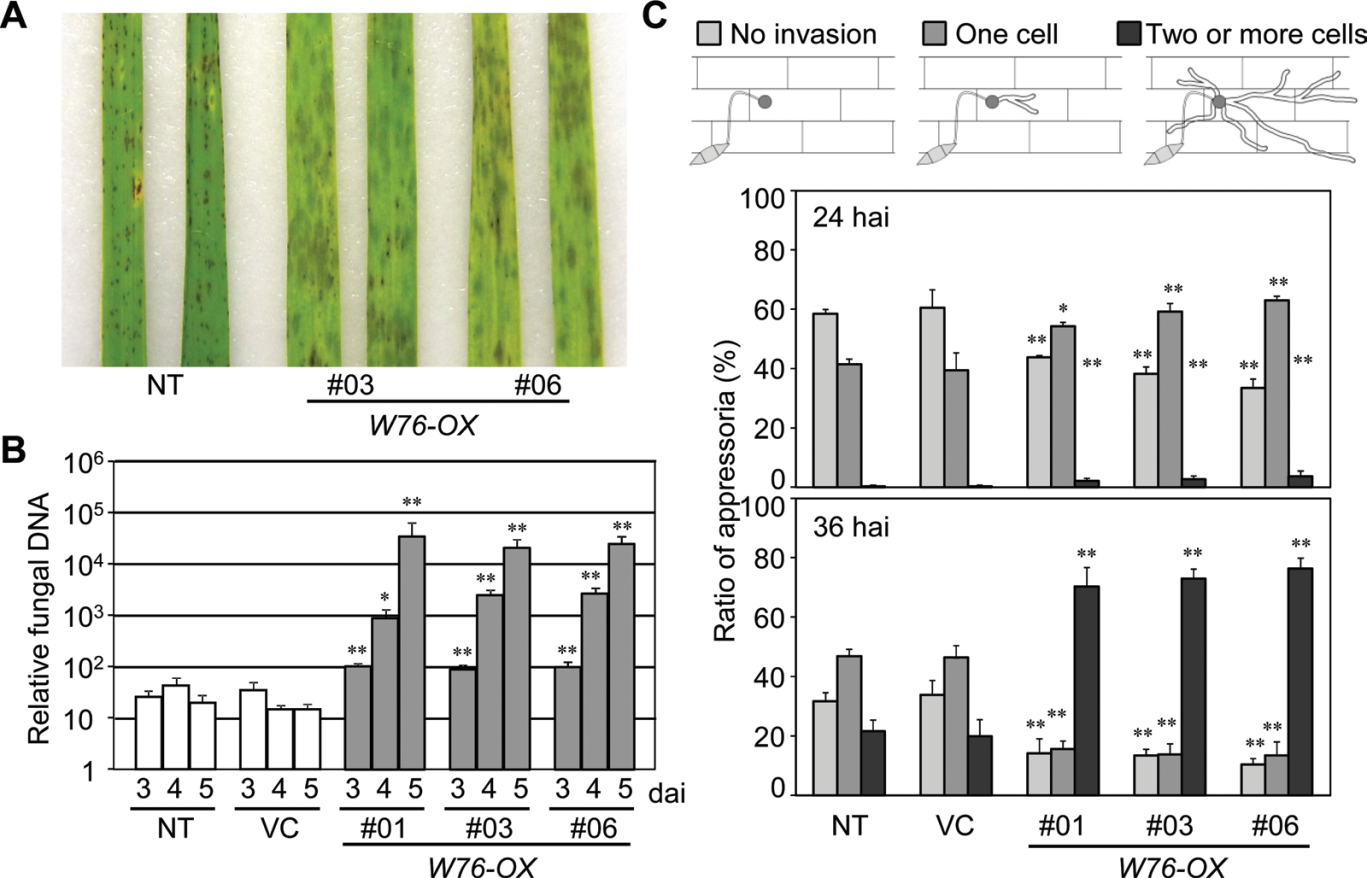
Effects of constitutive overexpression of *OsWRKY76* on susceptibility to a compatible strain of *M. oryzae*. (A) Disease symptoms on leaf blades at 4 dai. (B) Development of blast disease in leaf blades evaluated by quantitating *M. oryzae* genomic DNA. The amount of *M. oryzae* 28S rDNA relative to rice genomic *eEF1α* DNA was determined by quantitative PCR analysis. Values are represented as mean values ±SE for 12 leaf blades. Significantly higher values compared with those of the non-transformed control are denoted by asterisks (*P < 0.05, **P < 0.01 by Dunnett’s test on log-transformed data). (C) Invasion rate of the infectious hyphae in rice leaf sheath. Upper illustrations show the classification of patterns of invasion. Data are expressed as the ratio of the invasion pattern of infectious hyphae per 200 appressoria. Values are represented as mean values ±SE for five leaf sheaths. Significantly different values compared with those of the non-transformed control are denoted by asterisks (*P < 0.05, **P < 0.01 by Dunnett’s test).

The effect of overexpression of *OsWRKY76* on disease resistance to rice blast fungus was examined. Transgenic plants showed more severe symptoms, which often blasted the whole plant, compared with non-transformed control (NT) plants both in the excised leaves (Fig. 3A) and in the intact plants (Supplementary Fig. S3A at *JXB* online). Observations of cross-sections of the inoculated leaf blades revealed that in NT plants, the spread of mycelia was generally limited to the spaces between motor cells and vascular tissues at 4 dai. In *W76-OX* plants, however, the mycelia vigorously spread into parenchymatous tissues in addition to motor cells and vascular tissues, causing a collapse of the parenchyma, and hyphae already spread over the leaf surface at 4 dai (Supplementary Fig. S3B). Quantitative genomic PCR analysis demonstrated that >100-fold higher levels of fungal DNA were detected from *W76-OX* plants compared with NT plants (Fig. 3B). Microscopic observation of the inoculated leaf sheaths demonstrated that *W76-OX* plants allow penetration and elongation of infectious hyphae at significantly higher frequencies compared with NT plants (Fig. 3C; Supplementary Fig. S3C). In addition, *W76-OX* plants allowed greater penetration of an incompatible strain of *M. oryzae*, although the incompatibility was not overcome (Supplementary Fig. S4). These results indicate that the constitutive overexpression of *OsWRKY76* suppresses basal resistance to *M. oryzae* to a large extent.

### *Overexpression of* OsWRKY76 *improves tolerance to cold stress*

**Fig. 4.**
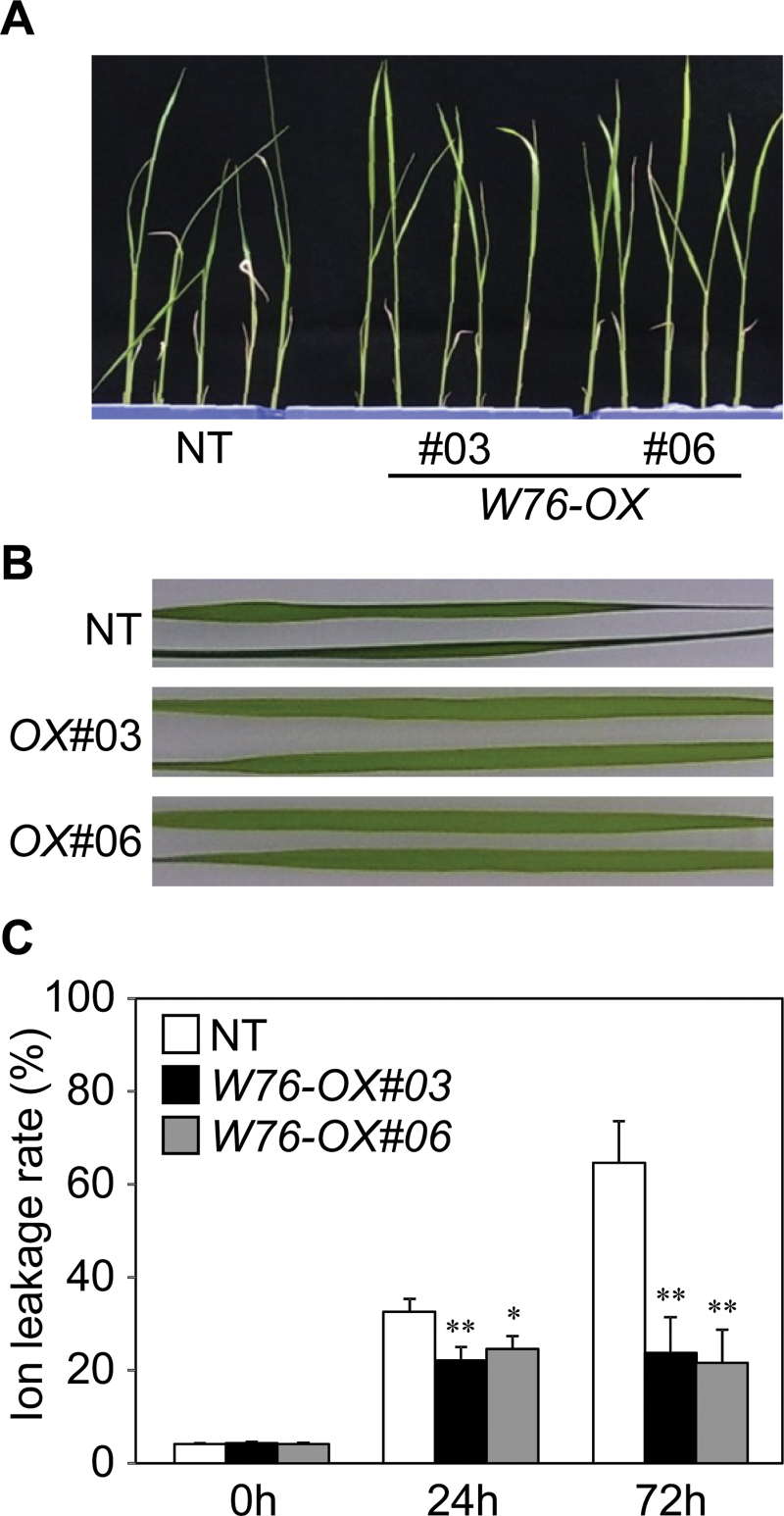
Tolerance of transgenic plants overexpressing *OsWRKY76* to cold stress. (A) Plants after treatment with cold stress. Fourteen-day-old plants were incubated at 4 °C in the dark for 72h. (B) Leaf blades after the cold treatment for 72h. (C) Ion leakage rate in the leaf blades after cold treatment. Values are represented as mean values ±SE for eight plants. Significantly lower values compared with the non-transformed control are denoted by asterisks (*P < 0.1, **P < 0.05 by Dunnett’s test). Experiments were carried out three times, and a representative result is shown.

The expression of *OsWRKY76* was induced by cold and ABA treatment as shown in Fig. 1. ABA mediates tolerance to low temperature in plants (Mauch-Mani and Mauch, 2005; Chinnusamy *et al.*, 2007); thus, the cold tolerance of *W76-OX* was examined. Following incubation at 4 °C for 72h, NT plants presented a severely wilted appearance, while *W76-OX* appeared normal (Fig. 4A, B). To determine the effect of cold stress on membrane stability, an ion leakage test was performed. After a 24h incubation at 4 °C, ion leakage from leaves had drastically increased, whereas in *W76-OX*, the cold-induced increase in ion leakage was significantly lower than that in the NT plants, and remained at similar levels even after 72h (Fig. 4C). Therefore, it is likely that constitutive overexpression of *OsWRKY76* confers cold tolerance to rice via protection of membranes from damage.

### *Genome-wide profiling of gene expression in* W76-OX

**Fig. 5.**
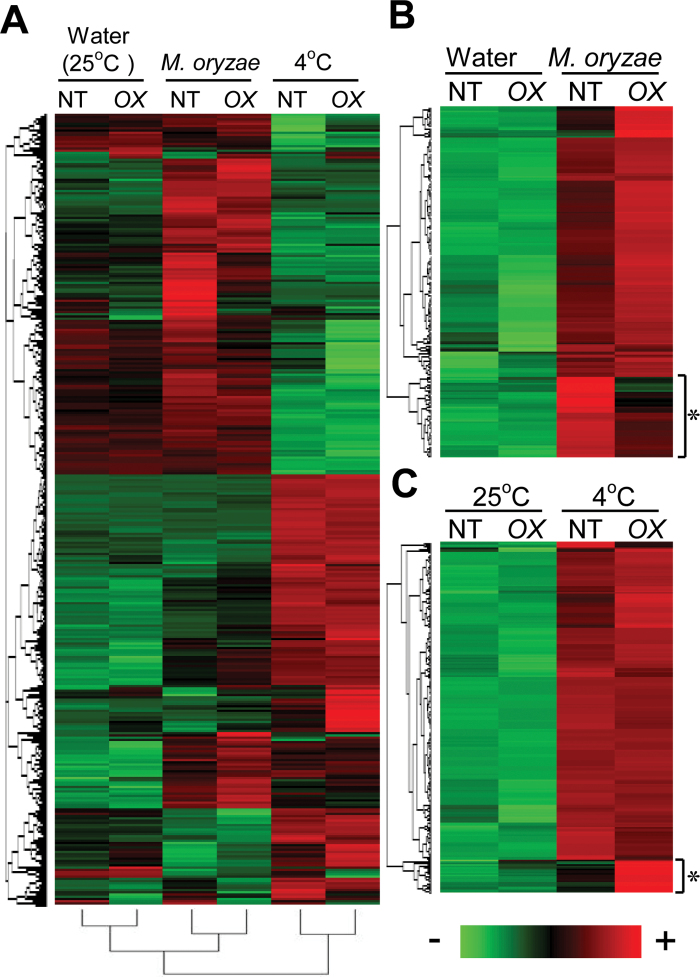
Gene expression profile of transgenic plants overexpressing *OsWRKY76* (*W76-OX#03*) in response to blast inoculation and cold stress. Lists of these genes are shown in Supplementary Tables S2, S3, and S4 at *JXB* online. (A) Result of microarray analysis presented in a heat map of z-scores. (B) Effect of *OsWRKY76* overexpression on the expression of blast disease-responsive genes in the leaf sheath at 36 hai. Genes with suppressed expression by *OsWRKY76* overexpression are indicated by a square bracket and an asterisk. (C) Effect of *OsWRKY76* overexpression on the expression of cold-responsive genes in the leaf blades incubated at 4 °C for 36h. Genes up-regulated by *OsWRKY76* overexpression are indicated by a square bracket and an asterisk.

To gain an insight into the role of *OsWRKY76* in blast susceptibility and cold tolerance, microarray analysis was performed. The fifth leaf sheaths of NT and *W76-OX* seedlings were inoculated with a compatible strain of *M. oryzae* or incubated at 4 °C for 36h. Leaf sheaths incubated at 25 °C for 36h were used as unstressed controls. After statistical processing, 1160 genes were selected by applying the filter of the average max/min difference being >6 (*P* < 0.01). An overview of a heat map with two-dimensional hierarchical clustering (Fig. 5A; Supplementary Table S2 at *JXB* online) revealed the following information. First, the number of genes that were up-regulated in response to inoculation of *M. oryzae* or cold treatment was larger than that of genes counteracted by these stresses. Secondly, a large number of genes showed opposing expression patterns between blast infection and cold treatment. Thirdly, gene expression profiles of NT and *W76-OX* plants in unstressed leaf sheaths were more similar to each other than those in blast-inoculated and cold-treated sheaths, indicating that the overexpression of *OsWRKY76* affects the expression of other genes upon exposure to these stresses. To identify the effect of *OsWRKY76* overexpression on the alteration of gene expression, 208 (Fig. 5B) and 338 (Fig. 5C) genes were next selected out of the 1160 genes that were at least 6-fold up-regulated by blast inoculation and cold treatment, respectively (*P* < 0.01) in either NT or *W76-OX* plants. A hierarchical clustering tree revealed the genes whose expression profiles were significantly different between NT and *W76-OX* plants. These genes might be involved in enhanced susceptibility to *M. oryzae* and tolerance to low temperature of *W76-OX* plants.

### *Overexpression of* OsWRKY76 *suppresses the activation of defence-associated genes*

**Fig. 6.**
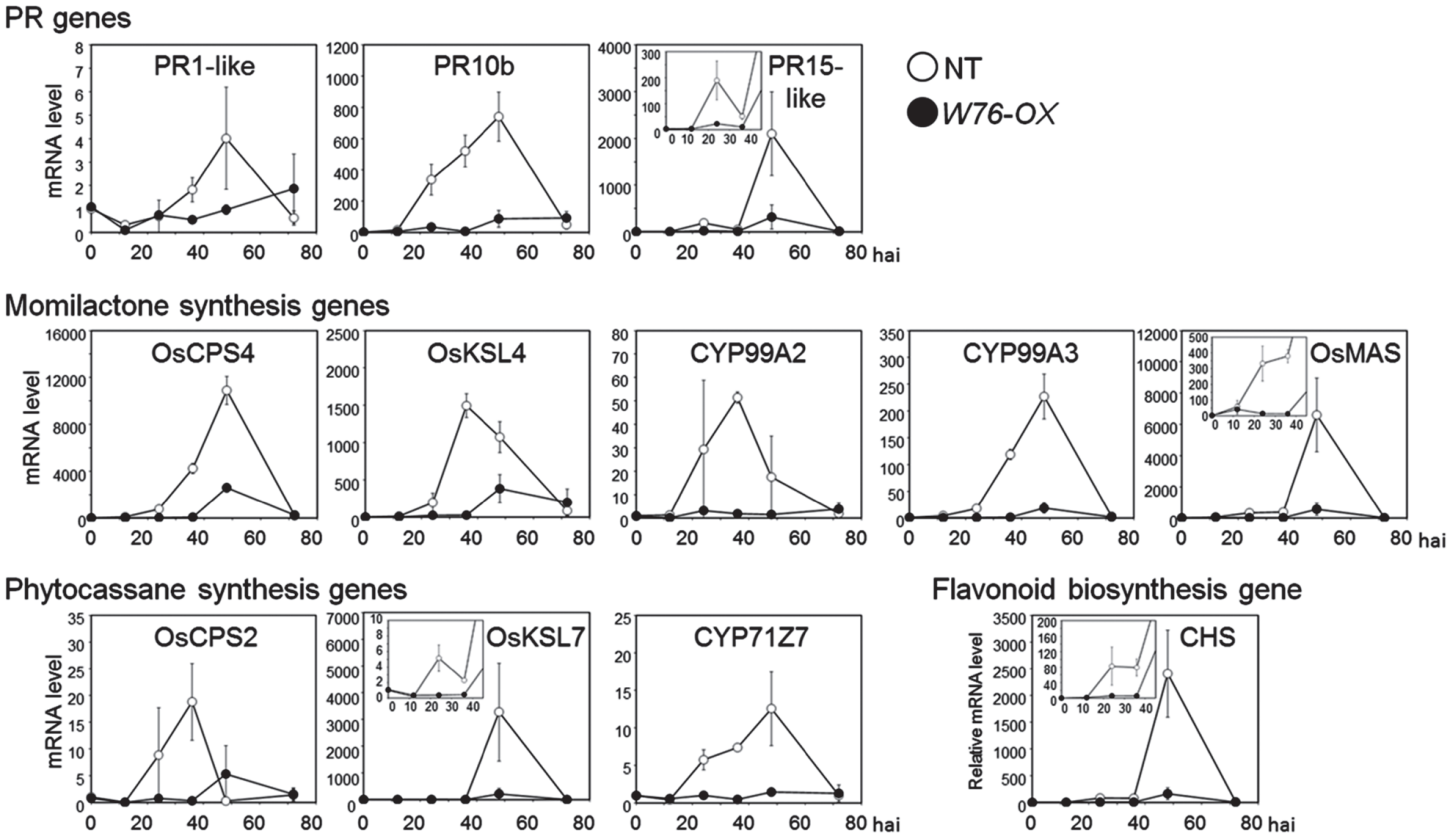
Effect of overexpression of *OsWRKY76* on the expression of PR genes and phytoalexin synthesis genes. Sheaths of the fifth leaves of NT and *W76-OX#03* were detached, inoculated with a suspension of conidia, and incubated at 25 °C in the dark. The time-course of gene expression was determined by qRT–PCR analysis. Transcription levels are expressed as the ratio to the level of transcript at 0h in NT. Data are represented as mean values ±SE for three replicates.

Among the 208 genes selected as up-regulated by blast inoculation either in NT or in *W76-OX* plants (Fig. 5B), 48 genes were counteracted in *W76-OX* plants, including several *PR* genes (Supplementary Table S3 at *JXB* online). To confirm the results of the microarray analysis further, qRT–PCR analysis was performed for *PR1*, *PR10b*, and *PR15*. As shown in Fig. 6, transcriptional induction of all these genes was drastically suppressed in *W76-OX* plants.

Another striking suppression was seen for genes encoding enzymes involved in phytoalexin biosynthesis. Two major classes of diterpenoid phytoalexins in rice, momilactones and phytocassanes, are synthesized from geranylgeranyl diphosphate (GGDP) through multiple steps (Supplementary Fig. S5 at *JXB* online), and multiple genes have been identified as those encoding the enzymes of momilactones and phytocassanes (Okada, 2011). The results of microarray analysis showed that, in *W76-OX* plants, the activation of five genes for momilactone synthesis (*OsCPS4*, *OsKSL4*, *CYP99A2*, *CYP99A3*, and *OsMAS*) and three genes for phytocassane synthesis (*OsCPS2*, *OsKSL7*, and *CYP71Z7*) was strongly suppressed (Supplementary Fig. S5); this was further confirmed by qRT–PCR in two independent *W76-OX* lines (Fig. 6; Supplementary Fig. S6). In addition, the blast-induced expression of the gene for chalcone synthase (CHS, Os04g0103900), one of the key enzymes in flavonoid synthesis, was also suppressed in *W76-OX* plants (Fig. 6; Supplementary Fig. S6).

### *Overexpression of* OsWRKY76 *reduces the blast-induced accumulation of phytoalexins*

**Fig. 7.**
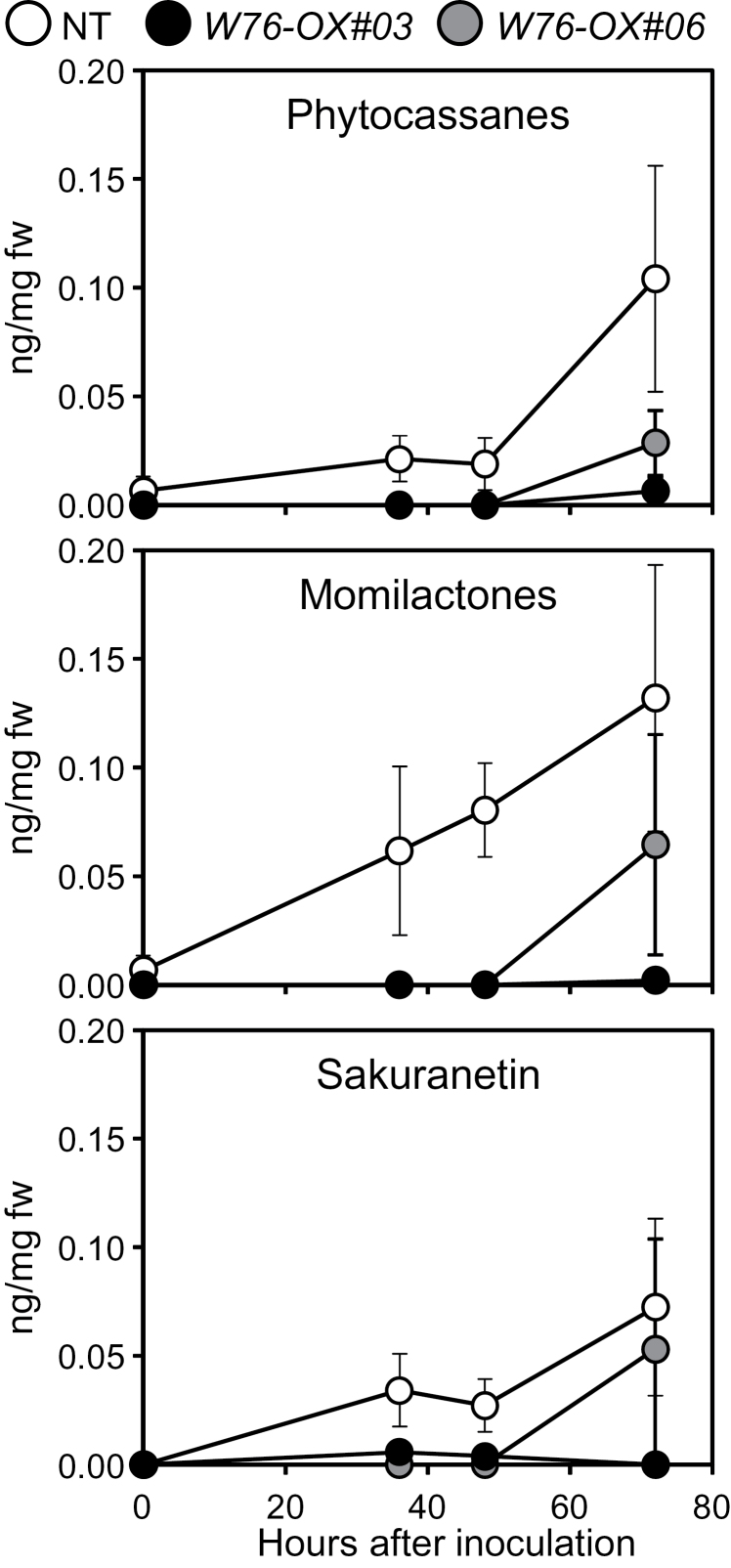
Effects of overexpression of *OsWRKY76* on the accumulation of phytoalexins in response to *M. oryzae* inoculation. The sheaths of the fifth leaves were detached, inoculated with a suspension of conidia, and incubated at 25 °C in the dark.

Next, the levels of the two classes of diterpenoid phytoalexins, phytocassanes and momilactones, and a flavonoid phytoalexin, sakuranetin, were determined in rice leaf sheaths after inoculation with the blast fungus. Accumulation of all three phytoalexins was observed at 36h after inoculation (hai) in NT plants, whereas in *W76-OX* plants, it was greatly suppressed (Fig. 7).

### Overexpression of *OsWRKY76* causes the up-regulation of abiotic stress-associated genes

Microarray analysis identified a total of 338 low temperature-responsive genes in either NT or *W76-OX* plants (Fig. 5C). Among them, 30 genes exhibited up-regulation, and five genes were counteracted in *W76-OX* plants in response to cold treatment (Supplementary Table S4 at *JXB* online). It was difficult to designate functions for the five counteracted genes, while the 30 up-regulated genes included those for antioxidant enzymes such as peroxidases (*OsPrx16/17*, *OsPrx39*, *OsPrx71*, and *OsPrx74*; Passardi *et al.*, 2007) and glutathione *S*-transferase (*OsGSTU5*; Soranzo *et al.*, 2004). Microarray analysis also detected three genes for lipid transfer proteins as up-regulated genes in *W76-OX* plants. Among these, Os04g0554800 and Os04g0644400 belong to the hybrid proline-rich protein (HyPRP) family and are homologues of *Arabidopsis EARLI1* (Bubier and Schläppi, 2004) and *AZI1* (Xu *et al.*, 2011), which confers cold tolerance to *Arabidopsis* possibly via the maintenance of membrane stability. The pollen development-related BURP domain gene *OsBURP13/OsRAFTIN1* (Wang *et al.*, 2003), which is very similar to the stress-associated gene *RD22* of *Arabidopsis* (Abe *et al.*, 1997), was also up-regulated in *W76-OX* plants.

**Fig. 8.**
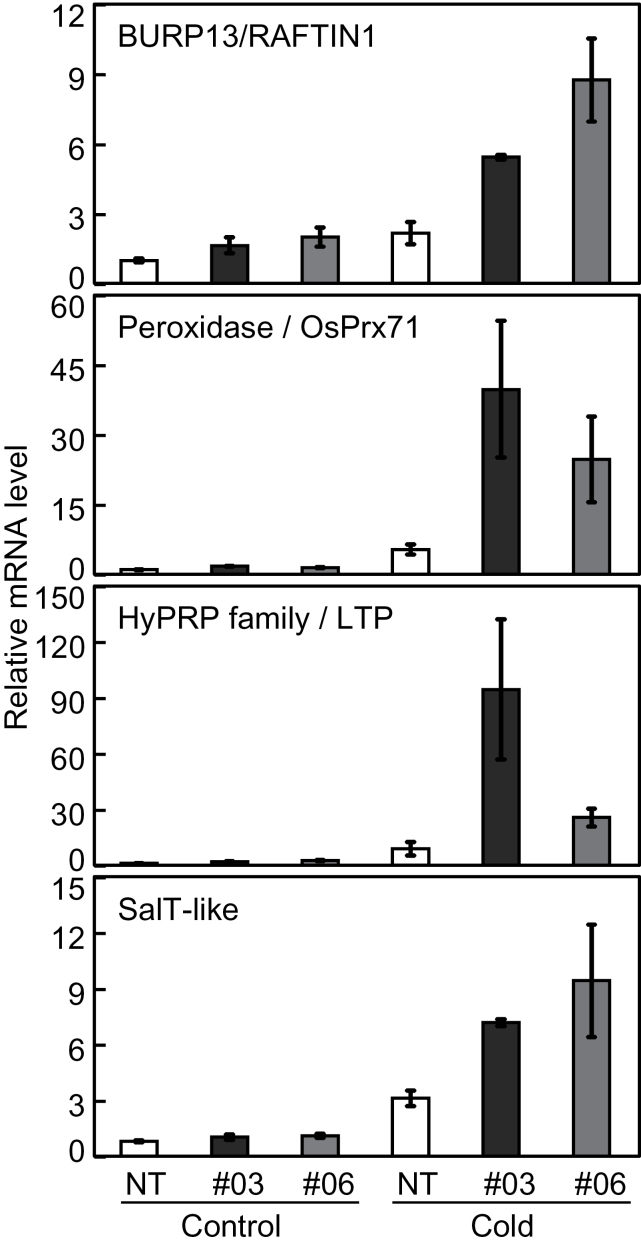
Effect of overexpression of *OsWRKY76* on the expression of cold stress-related genes. Transcription levels of NT and *W76-OX* lines were measured by qRT–PCR analysis. Data are expressed as the ratio to the level of transcript in NT at 25 °C.

QRT-PCR analyses were performed for *OsPrx71*, *OsBURP13/OsRAFTIN1*, a HyPRP family gene, and a *SalT*-like gene. As shown in Fig. 8, all the tested genes showed up-regulation in *W76-OX* plants following cold treatment.

## Discussion

The transcription factor *OsWRKY76*, a rice group IIa *WRKY* gene, was identified as a blast- and BTH-responsive gene (Ryu *et al.*, 2006; Shimono *et al.*, 2007). The results presented here clearly show that OsWRKY76 is a transcriptional repressor involved in both fungal disease resistance and cold stress tolerance. OsWRKY76 has two putative nuclear localization signals and a DNA-binding domain, but does not have any known plant transcriptional repressor domains such as an EAR motif (Ohta *et al.*, 2001). Thus, it is unknown how OsWRKY76 suppresses the transcription of its target genes. Several studies identified the transcriptional regulator activity of group IIa WRKY proteins. *OsWRKY71* (Chujo *et al.*, 2008) and *OsWRKY28* (Chujo *et al.*, 2013), closely related paralogues of *OsWRKY76*, encode proteins that exhibit transcriptional repressor activity in cultured rice cells. On the other hand, Chen *et al.* (2010) tested the transcriptional regulator activity of three WRKYs of group IIa in *Arabidopsis* using stable transformants, and found that AtWRKY18 and 60 are transcriptional activators while AtWRKY40 is a transcriptional repressor. Cotton GaWRKY1 activates the promoter of the sesquiterpene phytoalexin synthesis gene *CAD1-A* in transgenic *Arabidopsis* (Xu *et al.*, 2004). Therefore, not all group IIa *WRKY* genes encode transcriptional repressors, although the experimental systems used to test the activity differ in each report.

Overexpression of *OsWRKY76* enhanced the susceptibility of rice plants to the blast fungus (Fig. 3), indicating that it is a negative regulator of disease resistance. Seo *et al.* (2011) also reported that the overexpression of *OsWRKY76* causes reduced resistance to bacterial leaf blight caused by *X. oryzae*. Similarly, overexpression of *OsWRKY62*, a closely related paralogue of *OsWRKY76*, also causes enhanced susceptibility to *X. oryzae* (Peng *et al.*, 2008). Delteil *et al.* (2012) and Chujo *et al.* (2013) showed that *OsWRKY28* also acts as a negative regulator of resistance to *M. oryzae*. On the other hand, among genes encoding WRKY transcriptional activators in rice, the overexpression of *OsWRKY45* (Shimono *et al.*, 2007), *OsWRKY53* (Chujo *et al.*, 2007), *OsWRKY31* (Zhang *et al.*, 2008), and *OsWRKY30* (Peng *et al.*, 2012) confers disease resistance. These results consistently suggest that disease resistance in rice is negatively and positively regulated by WRKY transcriptional repressors and activators, respectively.

Microarray analysis and qRT–PCR analysis revealed that, in *W76-OX* plants, the up-regulation of a set of genes for PR proteins and enzymes involved in phytoalexin biosynthesis was largely cancelled after inoculation with *M. oryzae*. Therefore, these genes might be direct or indirect targets of OsWRKY76. The promoter regions of these genes include the W-box and W-box-like sequences (Supplementary Fig. S7 at *JXB* online), and it was found that OsWRKY76 binds to a W-box in the *PR10b* promoter (data not shown). The expression of these genes is, therefore, implicated to be regulated by OsWRKY76 via the W-box sequence. In rice, the blast-responsive expression of several defence-associated genes including *PR10b* is mediated by the SA signalling pathway (Shimono *et al.*, 2007), implying that *OsWRKY76* participates in SA signalling. Interestingly, blast-responsive expression of genes encoding almost all of the biosynthesis enzymes of diterpenoid phytoalexins, momilactones and phytocassanes, was suppressed (Fig. 6). These genes are clustered on chromosomes 4 and 2, respectively, and exhibited synchronous expression patterns in response to *N*-acetylchitooligosaccharide elicitors (Okada *et al.*, 2007). A bZIP transcription factor, OsTGAP1, is considered to regulate positively the expression of genes for diterpenoid phytoalexins (Okada *et al.*, 2009). However, because no alteration in *OsTGAP1* expression was observed in *W76-OX* plants, it is not clear at present how OsTGAP1 and OsWRKY76 cooperate with or counteract each other *in vivo* in the process of phytoalexin biosynthesis. Consistent with the expression profiles, accumulation of phytoalexins including sakuranetin was drastically reduced to low levels in *W76-OX* plants (Fig. 7). Because exogenously applied momilactones were shown to be toxic to *M. oryzae* in rice leaves at physiological concentrations (Hasegawa *et al.*, 2010), the enhanced susceptibility to *M. oryzae* in *W76-OX* plants is possibly due to the suppressed accumulation of these phytoalexins. This idea is further supported by the observation that a knock-out line of *OsCPS4*, which encodes *syn*-copalyl diphosphate synthase responsible for the biosynthesis of momilactones and oryzalexin S, is more susceptible to the rice blast fungus (Toyomasu *et al.*, 2013). Recently, Shimizu *et al.* (2012) purified an enzyme from rice leaves that catalyses the *O*-methylation of naringenin chalcone, forming sakuranetin (NOMT; naringenin *O*-methyltransferase). Microarray analysis, however, indicated that the overexpression of *OsWRKY76* suppressed the expression of not *OsNOMT* but *CHS* (Os04g0103900). Because naringenin chalcone is a key precursor to a variety of flavonoids, this result implies that the overexpression of *OsWRKY76* results in the reduction of several flavonoids in rice cells. *OsWRKY13* has also been reported to activate genes involved in the flavonoid biosynthesis pathway and in resistance to *M. oryzae* (Qiu *et al.*, 2008).

The up-regulation of *OsWRKY76* upon infection with *M. oryzae* is paradoxical, because *OsWRKY76* is a negative regulator of disease resistance. One hypothesis is that *OsWRKY76*, which is induced after *OsWRKY45*, adjusts the intensity of defence responses in cooperation with positive regulators of defence to protect the plant from the damage caused by defence responses. For example, *OsWRKY76* might restrict the biosynthesis of diterpenoid phytoalexins in order to maintain the pool of the substrate, GGDP, which is also used as a substrate for other components such as carotenoids, tocopherols, and chlorophylls (DellaPenna and Pogson, 2006). To test this hypothesis, it would be necessary to compare the levels of these metabolites in *OsWRKY76-*overexpressing plants and *OsWRKY76* knock-out or knock-down plants. Despite their efforts, the authors have not succeeded in isolating *OsWRKY76* knock-out/down plants. Similar feedback-like responses in the defence response have been reported in other plant species. For example, an *Arabidopsis* ERF/AP2 transcriptional repressor, AtERF4, is transcriptionally up-regulated by bacterial disease but negatively regulates disease resistance (McGrath *et al.*, 2005). A zinc-finger transcriptional repressor, *ZCT*, of *Catharanthus roseus* is induced by an elicitor and negatively regulates elicitor-induced genes involved in alkaloid biosynthesis (Pauw *et al.*, 2004).

*W76-OX* plants showed improved tolerance to low temperature stress (Fig. 4), indicating that OsWRKY76 functions as a positive regulator of the cold tolerance. Similarly, transgenic grass overexpressing *HvWRKY38*, a predicted orthologue of *OsWRKY76* in barley, shows improved tolerance to drought (Xiong *et al.*, 2010). Overexpression of *OsWRKY76* altered cold-induced electron leakage (Fig. 4C), which is a major problem causing chilling injury (Chinnusamy *et al.*, 2007). Microarray analysis revealed that a gene encoding a lipid transfer protein of the HyPRP family, which is predicted to play a role in membrane stability (Bubier and Schläppi, 2004; Xu *et al.*, 2011), was up-regulated by the overexpression of *OsWRKY76* (Fig. 8). In *W76-OX* plants, genes encoding defence-related proteins such as the antioxidant enzyme peroxidase were also up-regulated (Fig. 8). It might be possible that the up-regulation of these genes resulted in increased tolerance to cold treatment in *W76-OX* plants. Because OsWRKY76 shows transcriptional repressor activity (Fig. 2C), it is unlikely that OsWRKY76 directly activates the expression of these cold-responsive genes; instead, it may suppress the expression of negative regulators for these genes. Alternatively, the function of OsWRKY76 might be modulated to act as an activator by interaction with other proteins. There are reports that AtWRKY18, AtWRKY40, and AtWRKY60 form both homocomplexes and heterocomplexes, and function as transcriptional activators depending on the complex (Xu *et al.*, 2006; Chen *et al.*, 2010).

The present results demonstrate that OsWRKY76 plays opposite roles in blast resistance and cold tolerance when overexpressed. Low temperature treatment enhances the susceptibility of rice to *M. oryzae*, and, in fact, blast disease causes more serious damage in cold summer in the northern part of Japan (Matsuyama and Dimond, 1974; Koga *et al.*, 2004). Recent studies have suggested that the level of endogenous ABA is elevated by incubation at low temperature (Cuevas *et al.*, 2008), and low temperature-induced susceptibility to *M. oryzae* is mediated by ABA, which antagonizes SA signalling (Mauch-Mani and Mauch, 2005; Robert-Seilaniantz *et al.*, 2007; Ton *et al.*, 2009; Jiang *et al.*, 2010; Sharma *et al.*, 2013). A similar antagonistic interaction between SA and ABA has been observed in *Arabidopsis* (Yasuda *et al.*, 2008). *OsWRKY76* is transcriptionally up-regulated by treatment with BTH, a chemical activator of SA signalling, and ABA (Fig. 1), implying its involvement in the SA and ABA responses. OsWRKY76 might be involved in antagonistic regulation between SA-mediated biotic and ABA-mediated abiotic stress responses. Some studies suggest that WRKY might be associated with ABA signalling in several aspects (Rushton *et al.*, 2010). For example, *AtWRKY18*, *40*, and *60*, possible orthologues of *OsWRKY76* in *Arabidopsis*, have been suggested to be involved in ABA-associated stress responses (Chen *et al.*, 2010; Shang *et al.*, 2010).

In this study, it was demonstrated that OsWRKY76 plays important roles in plant responses to biotic and abiotic stresses. However, the signalling pathways upstream and downstream of OsWRKY76 remain to be elucidated. In *W76-OX* plants, the expression of genes encoding stress-associated signal components, including several types of transcriptional regulators, was also affected (Fig. 5; Supplementary Table S2 at *JXB* online), suggesting that responses to exogenous stresses are regulated by complex transcription events. Further analysis of these genes should be valuable for the purpose of balancing disease resistance and cold tolerance.

## Supplementary data

Supplementary data are available at *JXB* online.

Figure S1. Gel mobility shift competition assay of OsWRKY76 and the W-box-containing oligonucleotide.

Figure S2. Growth of transgenic plants constitutively overexpressing *OsWRKY76*.

Figure S3. Effects of overexpression of *OsWRKY76* on disease resistance to a compatible strain (Ina86-137) of *M. oryzae*.

Figure S4. Effects of overexpression of *OsWRKY76* on disease resistance to an incompatible strain (P91-15B) of *M. oryzae*.

Figure S5. Schematic view integrating the biosynthetic pathway of diterpenoid phytoalexins with microarray data.

Figure S6. Effects of the overexpression of *OsWRKY76* on the accumulation of phytoalexins in response to *M. oryzae* inoculation.

Figure S7. Distribution of W-box/W-box-like elements upstream (2kb) of the initiation codon of genes that are putatively regulated by OsWRKY76.

Table S1. Primers used for real-time PCR analysis.

Table S2. List of genes with significantly different expression profiles in *W76-OX* plants identified by microarray analysis (Fig. 5A).

Table S3. List of blast disease-responsive genes in the leaf sheath at 36 hai.

Table S4. List of cold-responsive genes in the leaf blades incubated at 4 °C for 36h.

Supplementary Data
