# Supplementary material for: WRKY76 is a rice transcriptional repressor playing opposite roles in blast disease resistance and cold stress tolerance
Source: J Exp Bot. 2013 Sep 16;64(16):5085–97. doi: 10.1093/jxb/ert298 (PMC3830488; doi:10.1093/jxb/ert298)
Supplement: Supplementary Data [file supp_64_16_5085__index.html]

OsWRKY76 is a rice transcriptional repressor playing opposite roles in blast disease resistance and cold stress tolerance — WRKY76 is a rice transcriptional repressor playing opposite roles in blast disease resistance and cold stress tolerance — Supplementary Data 

# WRKY76 is a rice transcriptional repressor playing opposite roles in blast disease resistance and cold stress tolerance

## Supplementary Data

Data files

**Files in this Data Supplement:**

- Supplementary Data - Supplementary Data
- Supplementary Data - Supplementary Data
