# Supplementary material for: WRKY76 is a rice transcriptional repressor playing opposite roles in blast disease resistance and cold stress tolerance
Source: J Exp Bot. 2013 Sep 16;64(16):5085–97. doi: 10.1093/jxb/ert298 (PMC3830488; doi:10.1093/jxb/ert298)
Supplement: Supplementary Data [file supp_ert298_jexbot099929_file002.pdf]

Supplementary Figures

OsWRKY76 is a rice transcriptional repressor playing opposite roles in blast disease resistance and cold stress tolerance

Running title:

OsWRKY76 in Biotic and Abiotic Stress Responses

Authors:

Naoki Yokotani[1], Yuko Sato[1], Shigeru Tanabe[1], Tetsuya Chujo[2], Takafumi Shimizu[2], Kazunori Okada[2], Hisakazu Yamane[3], Masaki Shimono[1], Shoji Sugano[1], Hiroshi Takatsuji[1], Hisatoshi Kaku[4], Eiichi Minami[1] and Yoko Nishizawa[1]

Address:

[1]Disease Resistant Crops Research Unit, GMO Research Center, National Institute of Agrobiological Sciences, 2-1-2 Kannondai, Tsukuba, Ibaraki 305-8602, Japan

[2]Biotechnology Research Center, The University of Tokyo, 1-1-1 Yayoi, Bunkyo-ku, Tokyo, 113-8657, Japan

[3]Department of Biosciences, Teikyo University, 1-1 Toyosatodai, Utsunomiya, Tochigi, 320-8551, Japan

[4]Sakata Seed Corporation, Kimitsu Research Station, 358 Uchikoshi, Sodegaura, Chiba 299-0217, Japan

Protein	M	W76	W76	W76	W76	W76	W76	W76
Probe	WB	WB	WB	WB	WB	WB	WB	WB
Competitor	-	-	WB x10	WB X100	WB X1000	mWB X10	mWB X100	mWB X1000

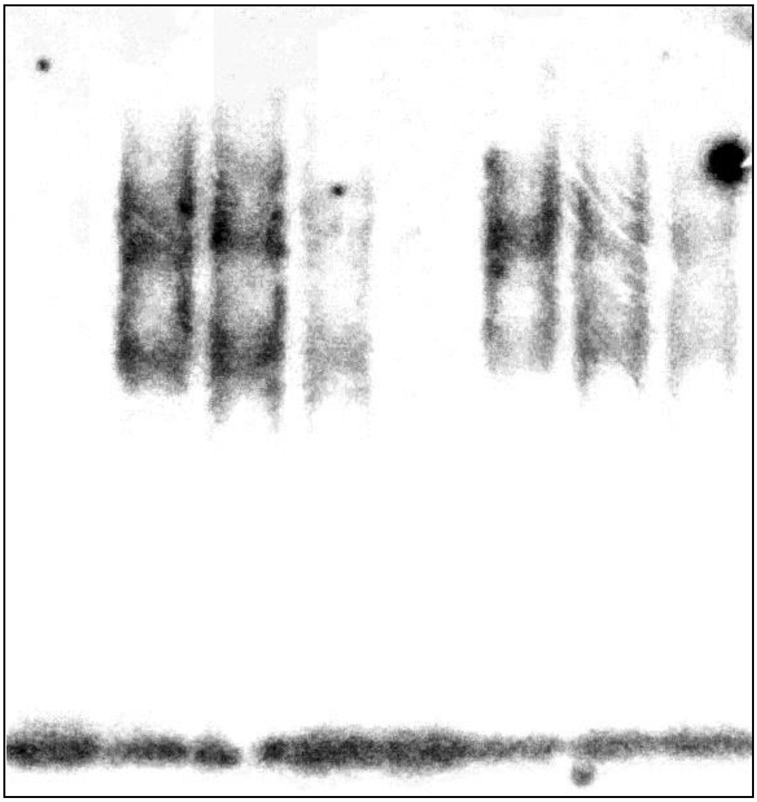

Supplemental Fig. S1. Gel mobility shift competition assay of OsWRKY76 to the W-box-containing oligonucleotide. Recombinant OsWRKY76 protein fused to MBP (W76) and MBP control (M) were used. Gel mobility shift by the reaction of recombinant OsWRKY76 protein and the probe containing W-box sequence was blocked by an excess of WB but not by mWB.

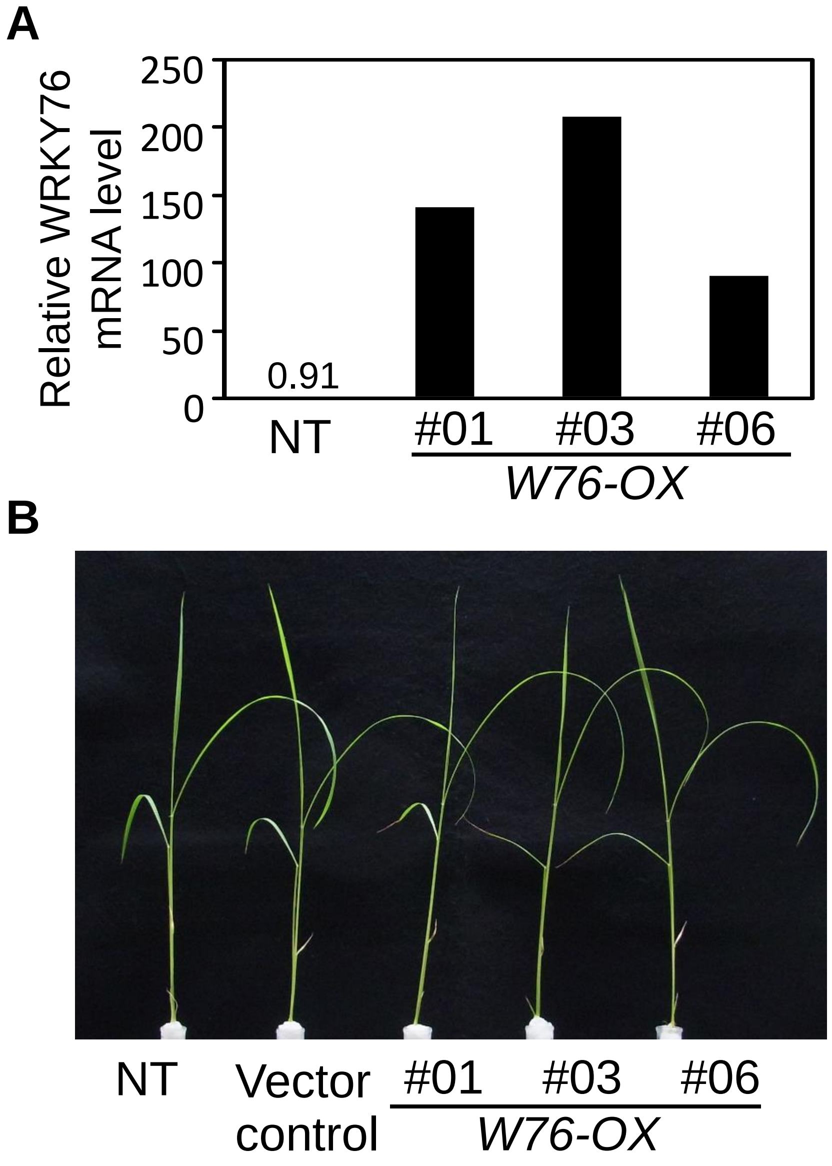

Supplemental Fig. S2. Growth of transgenic plants constitutively overexpressing *OsWRKY76*.
(A) Expression of *OsWRKY76* in non-transformed (NT) and transgenic plants. Expression levels were determined by quantitative RT-PCR analysis.
(B) Plants at the 5.6-leaf stage.

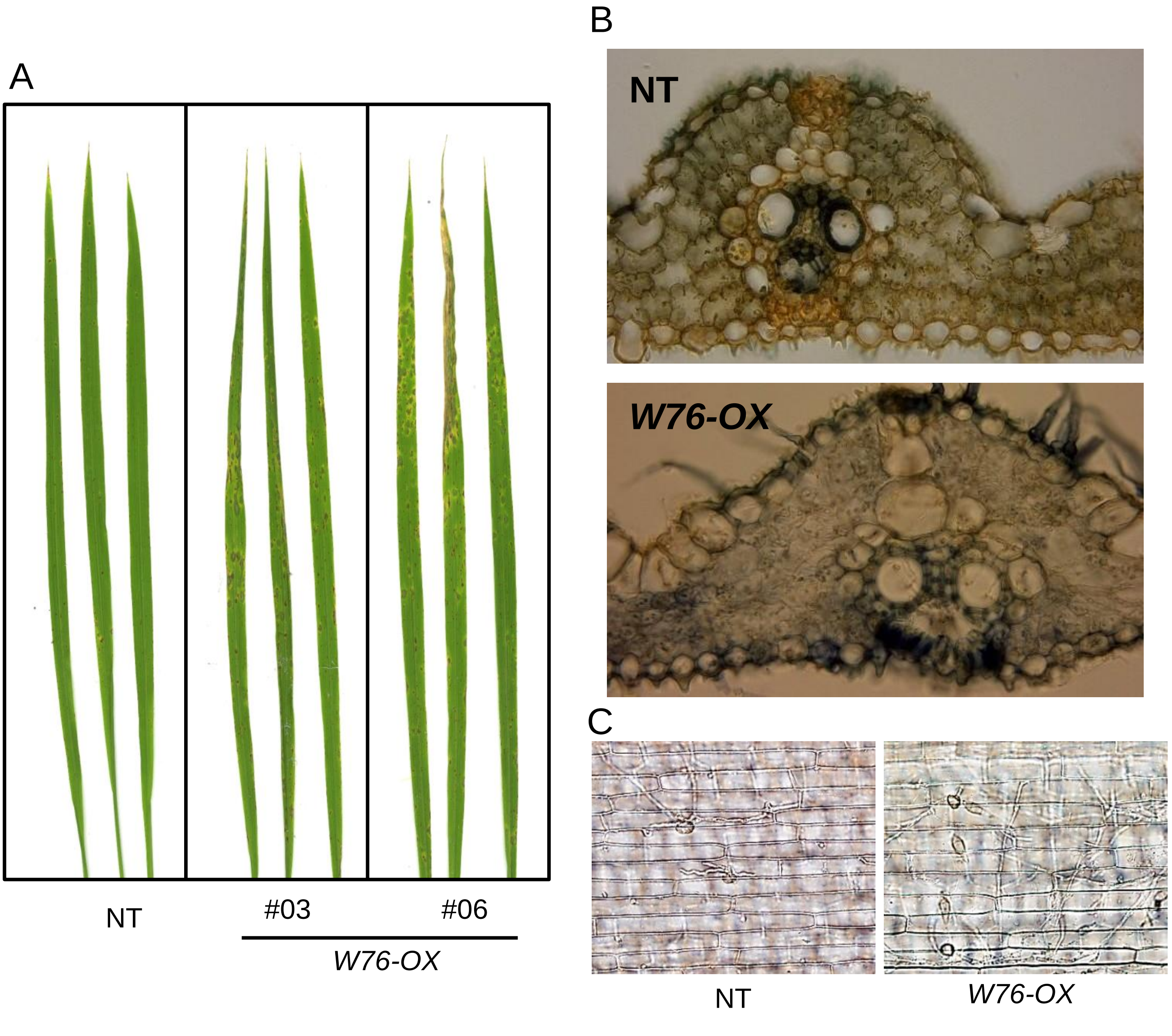

Supplemental Fig. S3. Effects of overexpression of *OsWRKY76* on disease resistance to a compatible strain (Ina86-137) of *M. oryzae*.

(A) Disease symptoms in the intact plants. Rice seedlings at the 5-leaf stage were sprayed with conidia suspended in water and incubated for 5 days.

(B) Photomicrographs of cross-sections of rice leave blades from inoculated intact plants. *W76-OX* plants show enhanced susceptibility to *M. oryzae* (4 dai).

(C) Microscopy of infectious hyphae in rice leaf sheath. Sheaths of the fifth leaves were detached, inoculated with a suspension of conidia, and incubated for 36 h in the dark.

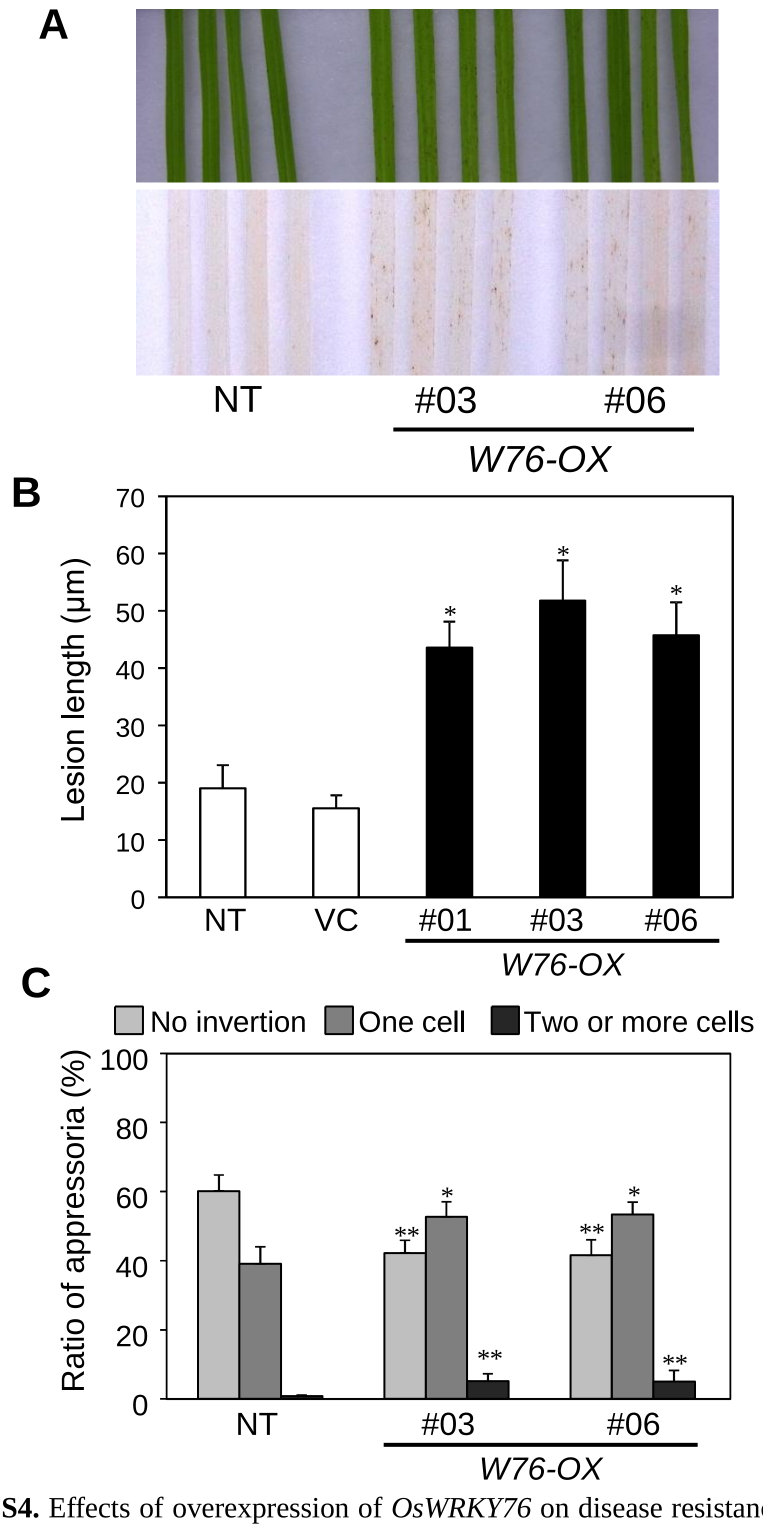

Supplemental Fig. S4. Effects of overexpression of *OsWRKY76* on disease resistance to an incompatible strain (P91-15B) of *M. oryzae*.

(A) Disease symptoms of plants. Fifth leaf blades detached from the rice plants were sprayed with a conidial suspension and incubated for 4 days. The lower photograph shows bleached leaf blades boiled in 100% ethanol.

(B) Lengths of lesions in leaf blades at 4 days after inoculation. Values are represented as mean values ± standard error (SE) value for the 20 replicates. Significantly lower values compared with those of the non-transformed control are denoted by asterisks (**p* < 0.01 by Dunnett’ s test).

(C) Invasion rate of the infectious hyphae in the rice leaf sheath. Patterns of infectious hyphae are shown in Figure 3C. Data are expressed as invasion rate of infectious hyphae per 200 appressoria. Values are represented as mean values ± SE for 5 leaf sheaths. Significantly different values compared with the non-transformed control are denoted by asterisks (**p* < 0.05, ***p* < 0.01 by Dunnett's test).

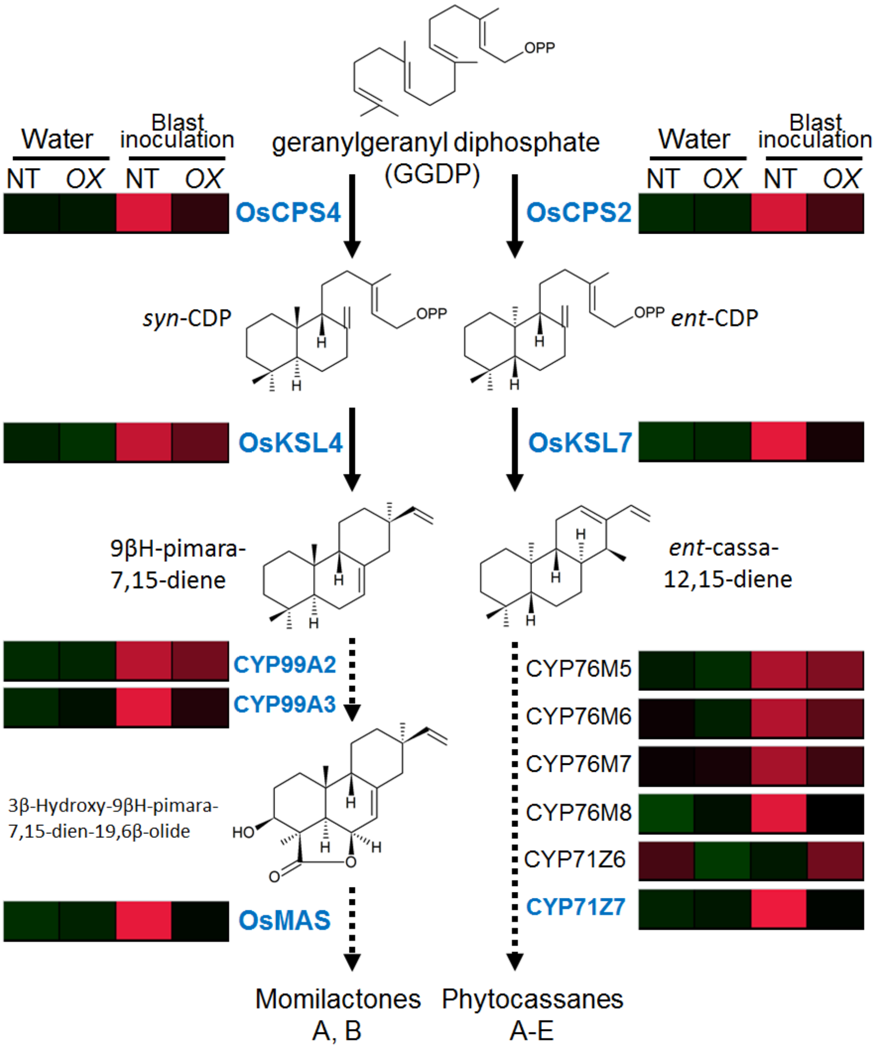

Figure S5. Schematic view integrating the biosynthetic pathway of diterpenoid phytoalexins with microarray data. Genes with significantly suppressed expression upon *OsWRKY76* overexpression (classified in a square bracket in Fig. 5B) are highlighted in blue. CDP, copalyl diphosphate; CPS, copalyl diphosphate synthase; KSL, kaurene synthase-like; MAS, momilactone A synthase; CYP, cytochrome P450 monooxygenase.

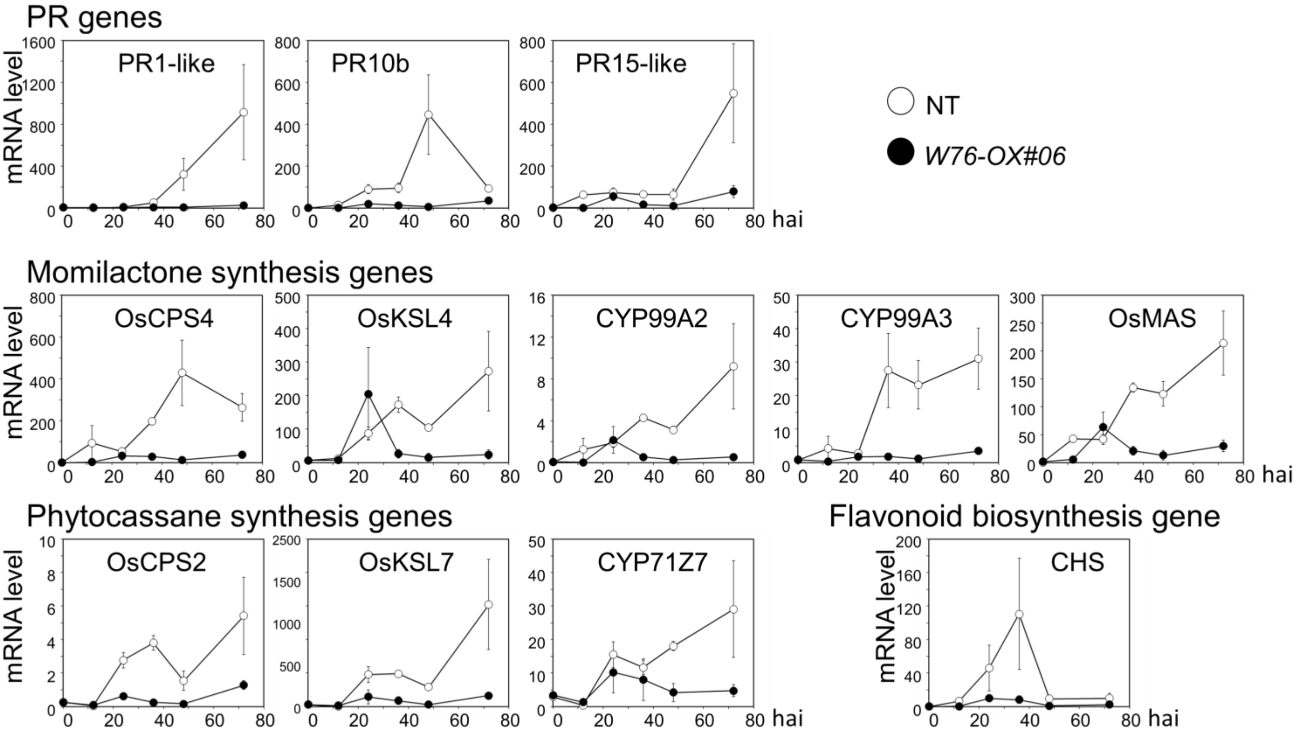

Supplemental Fig. S6. Effects of the overexpression of *OsWRKY76* on the accumulation of phytoalexins in response to *M. oryzae* inoculation. The sheaths of the fifth leaves were detached from NT and *W76-OX#06* plants, inoculated with a suspension of conidia, and incubated at 25° C in the dark.

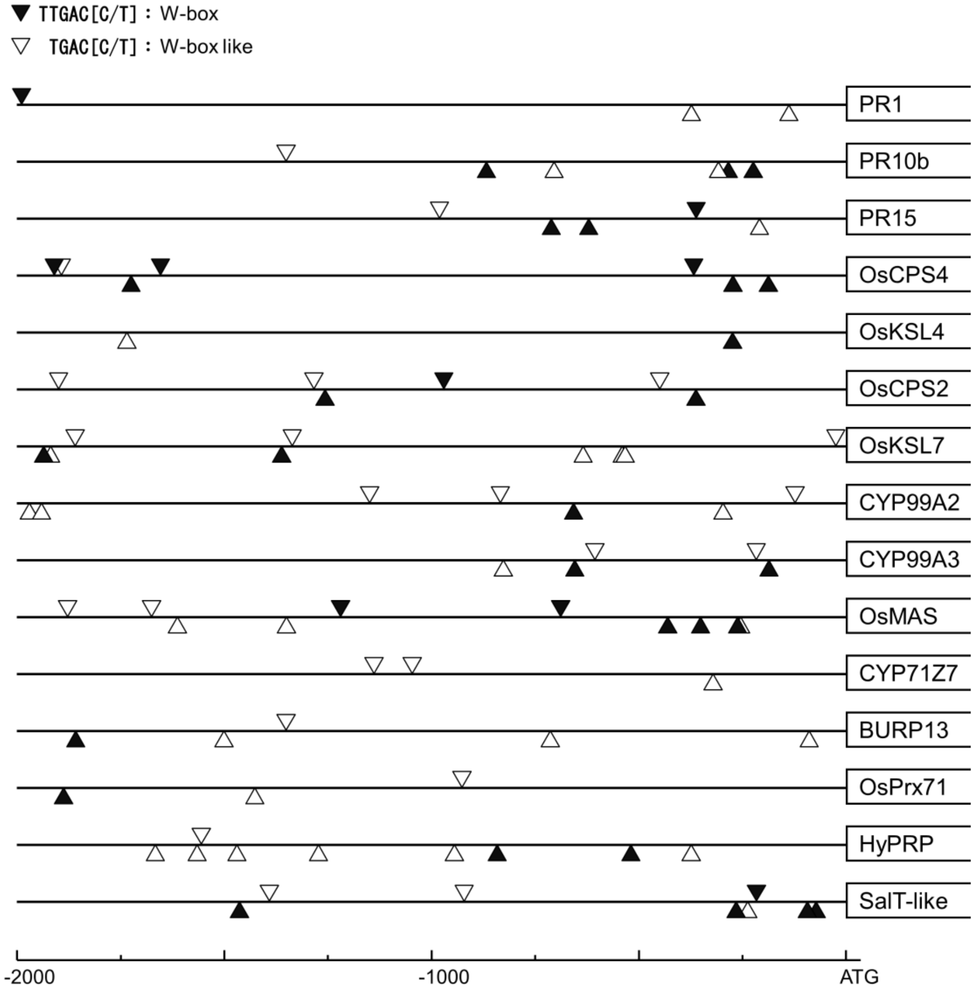

Supplemental Fig. S7. Distribution of W-box/W-box like elements in the upstream (2 kb) of the initiation codon of genes that are putatively regulated by OsWRKY76. Black and white triangles indicate the W-box and W-box-like elements, respectively.
